# Characteristics of Soil Microbial Community Structure in Different Land Use Types of the Huanghe Alluvial Plain

**DOI:** 10.3390/microorganisms13020273

**Published:** 2025-01-25

**Authors:** Xintong Cao, Qinghua Cui, Daiqing Li, Yu Liu, Kun Liu, Zhuoqing Li

**Affiliations:** 1 State Key Laboratory of Environmental Benchmarking and Risk Assessment, Chinese Research Academy of Environmental Sciences, Beijing 100012, China; cxt201230@163.com (X.C.); lidq@craes.org.cn (D.L.); lyu@craes.org.cn (Y.L.); 2 College of Ecology, Lanzhou University, Lanzhou 730000, China; liukun@lzu.edu.cn; 3 Institute of Environmental Protection Science and Technology of Binzhou, Binzhou 256600, China; qhcui1981@163.com

**Keywords:** soil microorganisms, land use types, metagenomic sequencing, community diversity

## Abstract

The Huanghe alluvial plain plays a crucial role in biodiversity conservation. However, its ecosystem has become sensitive and fragile due to long-term human disturbances. Enhancing the resilience of this ecosystem and promoting the sustainable use of land resources are key to addressing its ecological challenges. Soil microbial communities are vital to ecosystem functioning, and land use is a major human factor influencing their structure and diversity. Existing research on the Huanghe alluvial plain primarily focuses on soil physicochemical properties and moisture content, with relatively limited attention given to soil microorganisms. Therefore, this study, using the Wudi Tanyang Forest Farm in the Huanghe alluvial plain as a case study, employs high-throughput metagenomic sequencing to analyze the composition and diversity of soil bacteria, eukaryota, archaea, and virus communities in five different land use types (*Tamarix chinensis* forest, *Fraxinus chinensis* forest, farmland, wetland, and grassland). The results indicate that: (1) At the phylum level, the top three bacteria communities were Pseudomonadota, Acidobacteriota, and Actinomycetota; the top three in the eukaryota communities were Ascomycota, Mucoromycota, and Basidiomycotina; the top three in the archaea communities were Nitrososphaerota, Euryarchaeota, and Candidatus Thermoplasmatota; and the virus communities were dominated by Uroviricota; (2) The microbial community structure of the *Tamarix chinensis* forest and the *Fraxinus chinensis* forest was similar, and was significantly different from the other three land use types; (3) The land use type had a significant effect on the diversity of the soil microbial communities, with a higher diversity in the wetland and grassland soils; (4) The dominant species of the soil microbial communities under different land use types showed significant differences. This study provides theoretical support for land use optimization and sustainable soil management in the Huanghe alluvial plain region.

## 1. Introduction

Soil microorganisms are a crucial component of ecosystems, directly influencing plants by forming symbiotic and mutualistic interactions while also playing an essential role in nutrient biogeochemical cycling, promoting the decomposition of soil organic matter, and transforming nutrients. They act as a vital link between the above-ground and below-ground components of ecosystems [1,2]. The soil microbial community mainly includes bacteria, eukaryota, archaea, viruses, protozoa, and micro-animals. The two most important groups in the soil microbial community are soil bacteria and eukaryota, which exhibit global ecological niche differentiation in the surface soil [2]. Archaea are also a vital component of the soil microbial system and play a crucial role in soil biogeochemical cycles [3]. Viruses are ubiquitous in soil ecosystems, with concentrations of soil viruses reaching up to 109 VLPs (virus-like particles) per gram of dry soil [4]. Viruses can regulate host microbial communities in a top-down manner and can also directly or indirectly influence element cycling [5]. Research has demonstrated that soil microorganisms are fundamental to mineralizing organic matter, degrading pollutants, and promoting plant growth—all of which are vital for maintaining the soil ecosystem stability [6,7]. For instance, the phylum Nitrosococcales plays a key role in the soil nitrogen cycle, while Acidobacteria exhibit cellulolytic capabilities [8,9]. Šovljanski et al. [10] evaluated the combined effects of 11 operational and environmental factors on the microbiologically induced calcite precipitation (MICP) process, based on the concept that bacteria with a biomineralization capacity can effectively induce CaCO_3_ precipitation. They demonstrated that MICP can utilize the diversity of soil bacteria and emerge as an innovative bioremediation technique. Soil microorganisms exhibit significant species and genetic diversity, and due to their sensitivity to environmental changes, they can be influenced by shifts in the environment, vegetation, and biological interactions [11,12]. As such, soil microorganisms are considered key biological indicators for assessing soil ecological quality [13]. For example, Price et al. [14] conducted a study using a co-occurrence network analysis and a β-diversity analysis, and found that the structure and local diversity of soil microorganisms respond differently to environmental stress under varying frequencies and doses of alkaline-treated biosolid (ATB) application.

Land use encompasses various human activities (e.g., agriculture, forestry, and urbanization) that involve the development and utilization of soil resources to meet human economic or social needs, thereby significantly impacting soil ecosystems [15]. Changes in land use can alter soil-related ecological functions, thereby affecting the ecosystem stability and resource sustainability. At the community level, land use is recognized as one of the primary anthropogenic factors influencing soil microbial communities [16,17]. Shifts in land use typically affect the physical and chemical properties of the soil, nutrient cycling, and ecological functions, leading to changes in microbial communities [18,19]. For example, Tsiafouli et al. [20] conducted a study in Europe, which showed that land use changes due to intensive agriculture reduce soil microbial diversity. At the same time, soil microorganisms can adapt to land use changes by regulating the community structure and diversity levels, playing a key role in the functioning of ecosystems [7]. Zhou et al. [21] explored the impact of different agricultural management systems on crop species and soil microbial communities using the Illumina sequencing technology. The results indicated that organic management promoted healthier soils and supported more complex bacterial and fungal networks. Therefore, investigating the structural characteristics of soil microbial communities across different land use types is critical for understanding the geographical distribution of microbial communities in various soil types, identifying potential biological indicators of environmental change and gaining a deeper understanding of how soil and land use impact microbial diversity under different conditions.

Land use changes have significant impacts on ecosystems in different regions, and these impacts are particularly pronounced in the Huanghe alluvial plain. Due to its unique geographical, climatic, and human activity context, the region faces a series of distinctive ecological challenges. These challenges call for a more focused consideration of the vulnerability of the regional ecosystems and the need for sustainable management when studying land use. The Huanghe alluvial plain, located in China’s warm temperate zone, is a key area of interaction between the land and sea, which is rich in biodiversity and plays a significant role in biodiversity conservation. Compared with typical grassland or forest ecosystems, the hydrological system and wetland functions of the Huanghe alluvial plain play a unique and irreplaceable role in regulating the water cycle and providing habitats. The region’s agricultural production is closely integrated with its ecological functions, forming a distinctive “human–environment symbiosis” model that not only supports food production but also provides diverse ecosystem services. However, extensive irrigation and cultivation have altered the original ecological structure, leading to issues such as soil salinization, excessive groundwater extraction, and the reduction of the wetland areas [22,23]. As a result, ecological protection and restoration efforts, particularly the sustainable management of wetlands and water resources, have become crucial tasks for maintaining the region’s ecological functions. With the advancement of microbial research technologies, particularly molecular biology techniques, culture-based methods have been shown to be insufficient for accurately assessing soil microbial communities. In contrast, high-throughput sequencing technology, due to its high feasibility and cost-effectiveness, provides a more powerful and reliable means of evaluating microbial diversity, making it a crucial tool in the field of molecular microbial ecology [24,25]. In recent years, technologies such as metagenomics, metaproteomics, metatranscriptomics, and proteomics have gradually been widely applied in microbial ecology research [26]. The research by Das et al. [27] emphasizes the broad application of microbiomics in enhancing soil productivity by reviewing various sequencing platforms. The findings suggest that advances in modern ultra-high-throughput microbiomics, combined with cloud-based analytics, enable the in-depth exploration of soil microbial interactions, thereby promoting sustainable soil management and improving plant yield and productivity. Furthermore, metagenomic studies play a crucial role in revealing the abundance and diversity of microbial communities. The study area, the Wudi-Tanyang Forest Farm, is representative of the main land use types in the Huanghe alluvial plain, making it an ideal site for this research. Most studies on soils in this region have focused on their physical and chemical properties [28,29,30], and research on soil microorganisms is limited, with most studies concentrating primarily on soil bacteria [31,32] and no comprehensive investigations into the soil microbial communities, including archaea and viruses. This paper, therefore, uses metagenomic sequencing technology to examine the structural differences in soil bacteria, eukaryota, archaea, and virus communities under different land use types in the Huanghe alluvial plain. The aim is to provide a more comprehensive understanding of the soil microorganisms in the Huanghe alluvial plain, offering a theoretical foundation for addressing the ecological issues of the region and guiding future sustainable land use and soil management practices.

## 2. Materials and Methods

### 2.1. Sampling Site and Sample Collection

**Figure 1:**
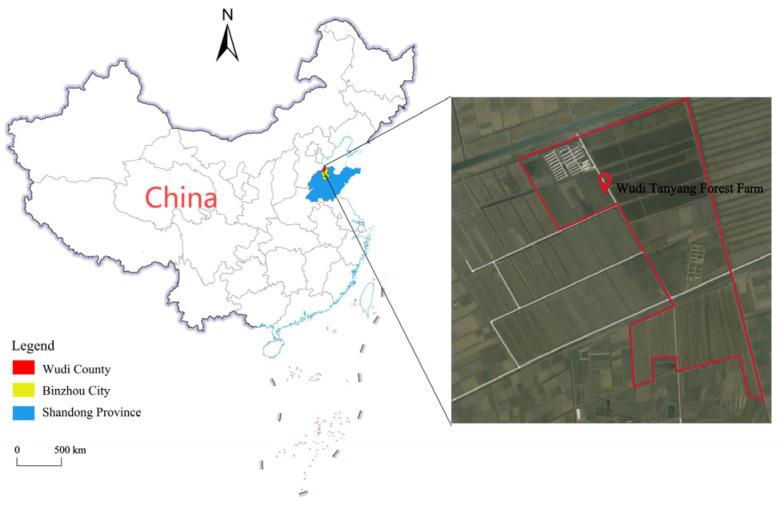
Sampling site and remote sensing image. Note: The base map is sourced from the National Standard Map Service website (https://bzdt.ch.mnr.gov.cn/index.html, accessed on 3 August 2023) and is a 1:10,000,000 political map of the People’s Republic of China. The review number is GS (2023) 2763. Download date: 3 August 2024.

Soil samples for this study were gathered in August 2023, at the Tanyang Forest Farm in Wudi County, Binzhou City, Shandong Province, China (37°40′ N~38°16′ N, 117°31′ E~118°04′ E) (Figure 1). The predominant soil type in this region is coastal tidal soil. August is the peak growing season for crops, during which microbial activity in the soil and the decomposition of organic matter are at their highest. We identified five primary land use types at Tanyang Forest Farm, namely *Tamarix chinensis* forest (TCF), *Fraxinus chinensis* forest (FCF), farmland (FL), wetland (WL), and grassland (GL), all of which had comparable slope, aspect, light radiation intensity, and soil preheating conditions. The *Tamarix chinensis* forest and *Fraxinus chinensis* forest were the two main forest types planted in the forestry farm. The *Tamarix chinensis* forest was dominated by *Tamarix chinensis* (*Tamaricaceae* family), while the *Fraxinus chinensis* forest was dominated by *Fraxinus chinensis* (*Oleaceae* family). These forests were classified as public welfare forests, with no management measures implemented after the initial fertilization and irrigation during the planting stage. The farmland was mainly used to grow corn, with irrigation, fertilization, and pesticide application during the growing season. The wetland was dominated by reed species (Phragmites australis) from the Gramineae family. The grassland was abandoned cropland that had been converted to fallow, with dominant species including Setaria viridis and Lolium perenne L., both of which belong to the Poaceae family. Five plots were selected for each land use type, for a total of 25 plots, based on the current conditions, vegetation types, and disturbance levels. Soil samples were collected using the five-point sampling method from a 0–10 cm depth with sterile shovels after removing the surface litter and plant debris, because the soil microbial activity is higher in the soil at this depth. The samples were packed into sterile self-sealing bags and transported to the laboratory at a low temperature for the soil microbial metagenomic sequencing analysis.

### 2.2. Metagenomic Sequencing

**Figure 2:**
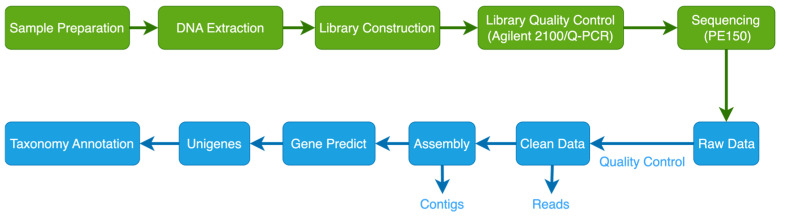
Metagenomic sequencing process.

The soil samples were transported to Beijing Novogene Technology Co., Ltd. (Beijing, China) at subzero temperatures for the metagenomic sequencing analysis. The analysis process is shown in Figure 2. Genomic DNA (gDNA) was extracted from 0.1 g of soil samples using the Soil Genomic DNA Kit (Jiangsu Cowin Biotech Co., Ltd., Taizhou, Jiangsu, China). The extracted DNA was assessed for quality using 1% agarose gel electrophoresis and precisely quantified with a Qubit 2.0 (ThermoFisher Scientific™, Waltham, MA, USA). The qualified gDNA was used to prepare sequencing libraries using the Next Ultra II DNA Library Prep Kit for Illumina (New England BioLabs Inc., Ipswich, MA, USA) per the manufacturer’s instructions. The initial quantification was performed using Qubit 2.0, and the concentration was diluted to 2 ng/μL to ensure library purity. The library’s insert fragments were analyzed using an Agilent 2100, and qPCR was performed to accurately quantify the library’s effective concentration (effective concentration > 3 nmol/L). The Illumina PE150 platform was used for sequencing once quality control was satisfied. Fastq was used to regulate the quality of the raw sequencing data and achieve high-quality reads [33]. The specific processing steps are as follows: (a) Remove reads containing low-quality bases (quality score ≤ 38) that exceed a certain proportion (40 bp); (b) Remove reads with a certain proportion of N bases (10 bp); (c) Remove reads with an overlap with adapters exceeding a certain threshold (15 bp); (d) Filter out reads originating from the host using the Bowtie2 software (http://bowtie-bio.sourceforge.net/bowtie2/index.shtml, accessed on 22 September 2023), with the parameters set to -end-to-end, -sensitive, -I 200, and -X 400. Data assembly was performed using the MEGAHIT1.2.9 software, selecting kmer values of 21, 29, 39, 59, 79, 99, 119, and 141 to obtain contigs. All contigs larger than 1500 bp were selected for further analysis. Gene prediction was performed using the MetaGeneMark software [34], followed by redundancy removal using the CD-HIT software. The longest sequences were then chosen as representative sequences to construct a non-redundant gene set, unigenes. These unigenes were aligned against bacteria, eukaryota, archaea, and virus sequences extracted from the NCBI NR database (https://www.ncbi.nlm.nih.gov/, accessed on 22 September 2023) using the DIAMOND2.1.10 software [35], and the LCA algorithm was used to determine species annotation. We used LCA annotation data and gene abundance tables to obtain abundance and gene count information for each sample at several taxonomic levels (kingdom, phylum, class, order, family, genus, and species). The abundance of a particular species in a given sample is equal to the sum of the gene abundances annotated as that species.

### 2.3. Statistical Analyses

We used the Shannon diversity index, the Simpson diversity index, the Chao1 index, and the Ace index to characterize the α-diversity of soil microorganisms. The Simpson, Shannon, Chao1, and Ace diversity indices can comprehensively reflect species diversity, evenness, and richness, and are applicable to various ecological and microbiological community studies. Among them, the Shannon index provides a comprehensive assessment of community complexity and stability; the Simpson index effectively measures community evenness and the influence of dominant species; while the Ace and Chao1 indices are effective for estimating potentially unobserved species, especially being more suitable for low-abundance species. A principal coordinate analysis (PCoA) with a Bray–Curtis distance was used to calculate the β-diversity, followed by the PERMANOVA test. The Lefse analysis was used to analyze the significantly different species. The primary data organization was carried out using the Excel 2021 software. All analyses and visualization were carried out in R4.3.2.

## 3. Results

**Figure 3:**
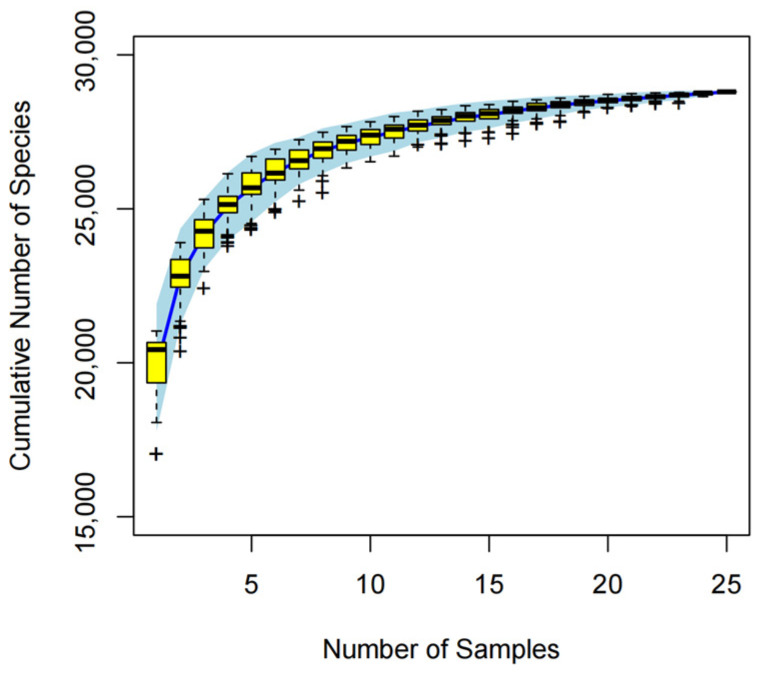
Soil microbial species accumulation curve.

To verify whether the soil microbial samples were sufficient, we constructed a species accumulation curve (Figure 3). The x-axis represents the number of samples, and the y-axis shows the number of newly detected species across all the samples. The results indicate that as the number of samples increases, the curve levels off, suggesting that additional samples contribute only a small number of new species. Therefore, it can be concluded that the sample size in this experiment is adequate.

The soil microbes in different land use types were classified and annotated using the KEGG database, resulting in 4 domains, 203 phyla, 200 classes, 406 orders, 948 families, 4186 genera, and 29,307 species. The proportions of bacteria, eukaryota, archaea, and virus annotated in the five land use types are shown below (Table 1), with bacteria being the most abundant, accounting for over 90% in all land use types.

### 3.1. Analysis of the Soil Bacteria Community Structure in Different Land Use Types

**Figure 4:**
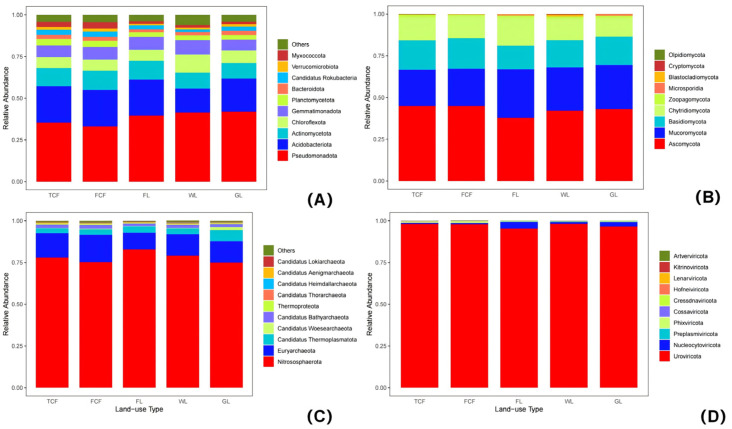
Analysis of the horizontal structure composition of the soil microorganisms phyla in different land use types: (**A**) bacteria, (**B**) eukaryota, (**C**) archaea, and (**D**) virus.

**Figure 5:**
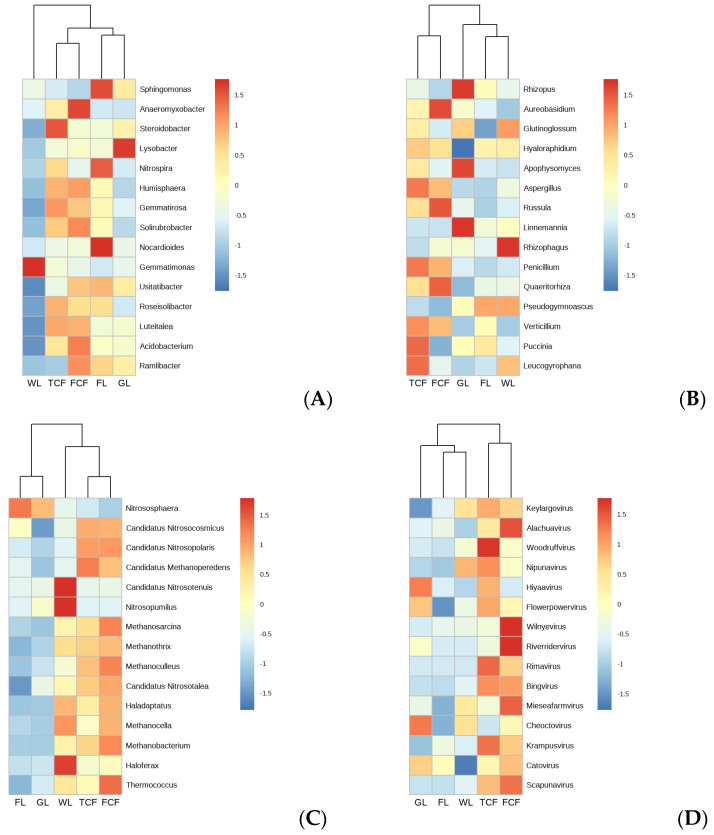
Cluster analysis of the soil microorganisms genera in different land use types: (**A**) bacteria, (**B**) eukaryota, (**C**) archaea, and (**D**) virus.

**Figure 6:**
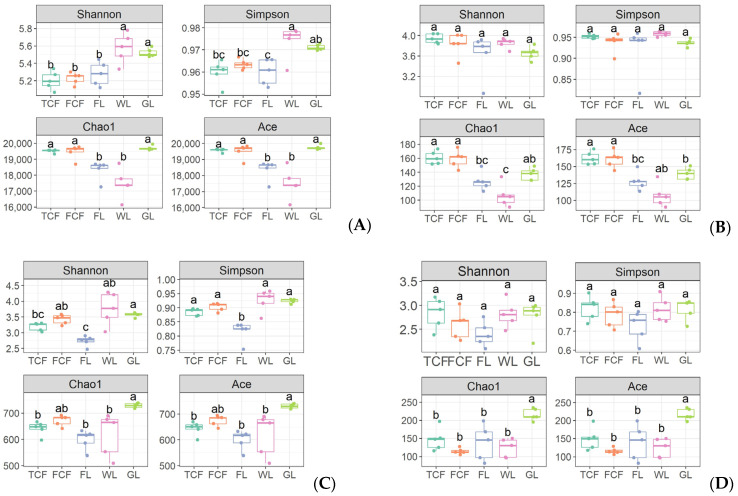
Analysis of the α-diversity of soil microorganisms in different land use types: (**A**) bacteria, (**B**) eukaryota, (**C**) archaea, and (**D**) virus. Different letters mean a significant difference at a 0.05 level.

**Figure 7:**
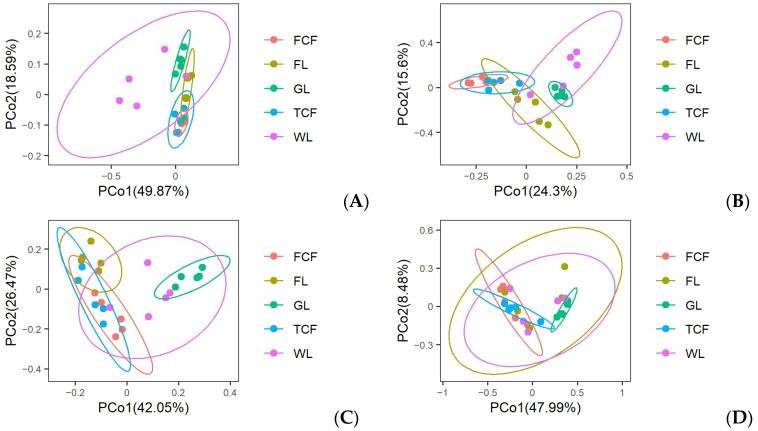
Distribution patterns of the β-diversity of soil microorganisms in different land use types: (**A**) bacteria, (**B**) eukaryota, (**C**) archaea, and (**D**) virus.

Through the structural analysis of the top 10 bacteria phyla by abundance (Figure 4A), it was found that the three most abundant bacteria phyla were Pseudomonadota (33.11–41.94%), Acidobacteriota (14.36–21.89%), and Actinomycetota (9.34–11.43%). At the genus level, a heatmap was generated for the top 15 most abundant genera (Figure 5A). The heatmap revealed that the soil bacteria communities of the *Tamarix chinensis* and *Fraxinus chinensis* forests initially clustered together, followed by the clustering of the farmland and grassland soil bacteria communities, and finally, the wetlands clustered last. This indicates that the bacteria communities in the soils of the *Tamarix chinensis* and *Fraxinus chinensis* forests, as well as those in the farmland and wetland, share more similar genus-level structures. The analysis of the Shannon diversity index, the Simpson diversity index, the Chao1 index, and the Ace index for the soil bacteria communities under the five land use types (Figure 6A) showed that the wetland had the highest Shannon and Simpson indices, which were significantly higher than those of the *Tamarix chinensis* forest, the *Fraxinus chinensis* forest, and the farmland (*p* < 0.05). The farmland and wetland had the lowest Chao1 and Ace indexes, and were significantly lower than the *Tamarix chinensis* forest, the *Fraxinus chinensis* forest, and the grassland (*p* < 0.05). The PCoA analysis based on Bray–Curtis distances showed that soil bacteria communities were differentiated across different land use types (Figure 7A). The PERMANOVA analysis (Table 2) indicated no significant difference in bacteria communities between the *Tamarix chinensis* and *Fraxinus chinensis* forests (*p* > 0.05), but significant differences were found between these two forest types and the other three land use types (*p* < 0.05).

**Figure 8:**
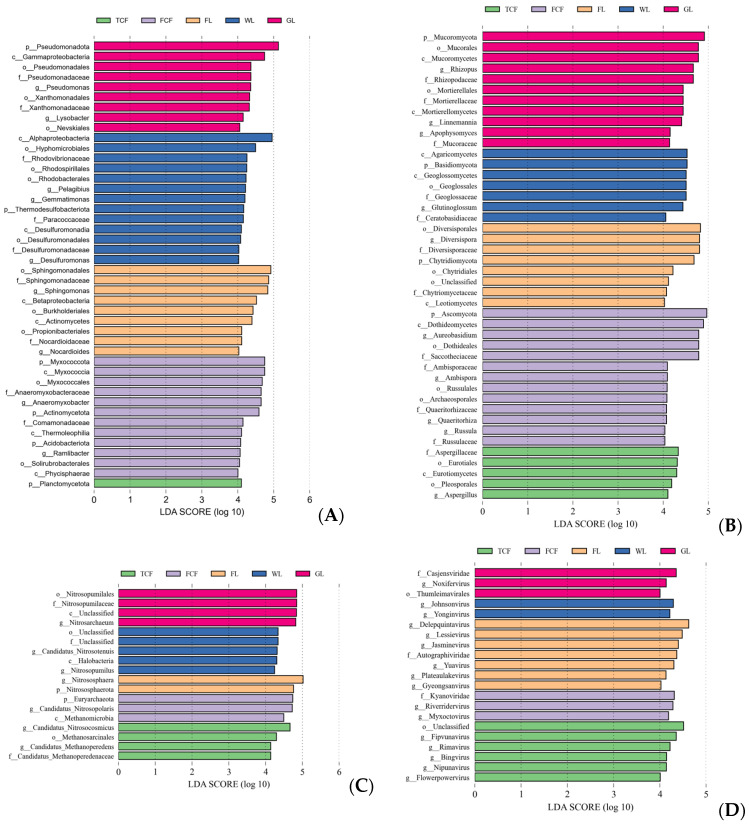
Lefse analysis of soil microorganisms in different land use types. An LDA score of 4.0 was used as a cut-off: (**A**) bacteria, (**B**) eukaryota, (**C**) archaea, and (**D**) virus.

The Lefse analysis results for the soil bacteria communities in different land use types are shown in Figure 8A. At the phylum, class, order, family, and genus levels, significant differences in dominant species were found among the five land use types. At the phylum level, Planctomycetota in *Tamarix chinensis* forest; Myxococcota, Actinomycetota, and Acidobacteriota in *Fraxinus chinensis* forest; Thermodesulfobacteriota in farmland; and Pseudomonadota in grassland were significant differences in land use types. At the class level, Myxococcia, Thermoleophilia, and Phycisphaerae in *Fraxinus chinensis* forest; Betaproteobacteria and Actinomycetes in farmland; Alphaproteobacteria and Desulfuromonadia in wetland; and Gammaproteobacteria in grassland were significant differences in land use types. At the order level, Myxococcales and Solirubrobacterales in *Fraxinus chinensis* forest; Sphingomonadales, Burkholderiales, and Propionibacterineae in farmland; Hyphomicrobiales, Rhodovibrionaceae, and Desulfuromonadales in wetland; and Pseudomonadales, Xanthomonadales, and Nevskiales in grassland were significant differences in land use types. At the family level, Anaeromyxobacteraceae and Comamonadaceae in *Fraxinus chinensis* forest; Sphingomonadaceae and Nocardioidaceae in farmland; Rhodoribvionaceae, Peptococcaceae, and Desulfuromonadaceae in wetland; and Pseudomonadaceae and Xanthomonadaceae in grassland were significant differences in land use types. At the genus level, Anaeromyxobacter and Ramlibacter in *Fraxinus chinensis* forest; Sphingomonas and Nocardioides in farmland; and Pelagibius, Gemmatimonas, Pseudomonas, and Desulfuromonas in wetland were significant differences in land use types.

### 3.2. Analysis of the Soil Eukaryota Community Structure in Different Land Use Types

The structural analysis of the top 10 eukaryota phyla, based on their abundance in soil eukaryota communities (Figure 4B), reveals that the three most abundant phyla are Ascomycota (37.77–44.95%), Mucoromycota (21.68–29.74%), and Basidiomycotina (14.17–18.20%). At the genus level, a heatmap of the top 15 most abundant eukaryota genera in the soil community (Figure 5B) shows that the soil eukaryota communities in the *Tamarix chinensis* and *Fraxinus chinensis* forests cluster together, as do those in the farmland and wetland; these then cluster with the grassland soil eukaryota communities. The analysis of the Shannon diversity index, the Simpson diversity index, the Chao1 index, and the Ace index of soil eukaryota communities under five land use types (Figure 6B) indicates no significant differences in the Shannon and Simpson indices (*p* > 0.05). However, the Chao1 and Ace indices are significantly higher in the *Tamarix chinensis* and *Fraxinus chinensis* forests than in the farmland (*p* < 0.05). A PCoA analysis based on Bray–Curtis distances shows differentiation in soil eukaryota communities in different land use types (Figure 7B). The PERMANOVA analysis results (Table 2) indicate significant differences (*p* < 0.05) in soil eukaryota communities in the five land use types.

The Lefse analysis of soil eukaryota communities under different land use types (Figure 8B) revealed significant differences in dominant species across the phylum, class, order, family, and genus levels. At the phylum level, Ascomycota in *Fraxinus chinensis* forest; Chytridiomycota in farmland; Basidiomycota in wetland; and Mucoromycota in grassland were significant differences in land use types. At the class level, Eurotiomycetes in *Tamarix chinensis* forest; Dothideomycetes in *Fraxinus chinensis* forest; Leotiomycetes in farmland; Agaricomycetes and Geoglossomycetes in wetland; and Mucoromycetes and Mortierellomycetes in grassland were significant differences in land use types. At the order level, Eurotiales and Pleosporales in *Tamarix chinensis* forest; Dothideales, Russulales, and Archaeosporales in *Fraxinus chinensis* forest; Diversisporales and Chytridiales in farmland; Geoglossales in wetland; and Mucorales and Mortierellales in grassland were significant differences in land use types. At the family level, Aspergillaceae in *Tamarix chinensis* forest; Saccotheciaceae, Micromonosporaceae, Quaeritorhizaceae, and Russulaceae in *Fraxinus chinensis* forest; Diversisporaceae and Chytriomycetaceae in farmland; Geoglossaceae and Ceratobasidiaceae in wetland; and Mortierellaceae and Mucoraceae in grassland were significant differences in land use types. At the genus level, Aspergillus in *Tamarix chinensis* forest; Aureobasidium, Ambispora, and Russula in *Fraxinus chinensis* forest; Diversispora in farmland; Glutinoglossum in wetland; and Rhizopus and Linnemannia in grassland were significant differences in land use types.

### 3.3. Analysis of the Soil Archaea Community Structure in Different Land Use Types

Through the structural analysis of the top 10 phylum-level abundances of soil archaea communities (Figure 4C), it can be observed that the three most abundant phyla in archaea are Nitrososphaerota (74.93–82.81%), Euryarchaeota (10.09–16.31%), and Candidatus Thermoplasmatota (2.86–6.64%). At the genus level, a heatmap of the top 15 most abundant genera in the soil archaea communities was generated (Figure 5C). The heatmap shows that the soil archaea communities in the farmland and grassland cluster together, while those in the *Tamarix chinensis* forest and *Fraxinus chinensis* forest form a separate cluster, which later merges with the grassland cluster. The analysis of the Shannon diversity index, the Simpson diversity index, the Chao1 index, and the Ace index of the soil archaea communities under five land use types shows (Figure 6C) that the grassland has higher Shannon, Simpson, Chao1, and Ace indices, which are significantly higher than the farmland (*p* < 0.05). The PCoA analysis based on the Bray–Curtis distance indicates differentiation in soil archaea communities in different land use types (Figure 7C). The PERMANOVA analysis results (Table 2) indicate that there is no significant difference between the *Tamarix chinensis* forest and the *Fraxinus chinensis* forest (*p* > 0.05), but significant differences exist between these two and the other three land use types (*p* < 0.05).

The Lefse analysis results of soil archaea communities in different land use types are shown in Figure 8C. At the phylum, class, order, family, and genus levels, significant differences in dominant species exist among the soil archaea communities of the five land use types. At the phylum level, Euryarchaeota in *Fraxinus chinensis* forest and Nitrososphaerota in farmland were significant differences in land use types. At the class level, Methanomicrobia in *Fraxinus chinensis* forest and Halobacteria in wetland were significant differences in land use types. At the order level, Methanosarcinales in *Tamarix chinensis* forest and Nitrosopumilales in grassland were significant differences in land use types. At the family level, Candidatus ethanoperedenaceae in *Tamarix chinensis* forest and Nitrosopumilaceae in grassland were significant differences in land use types. At the genus level, Candidatus Methanopereden and Candidatus Nitrosocosmicus in *Tamarix chinensis* forest; Candidatus Nitrosopolaris in *Fraxinus chinensis* forest; Nitrososphaera in farmland; Nitrosopumilus and Candidatus Nitrosotenuis in wetland; and Nitrosarchaeum in grassland were significant differences in land use types.

### 3.4. Analysis of the Soil Virus Community Structure in Different Land Use Types

Through the analysis of the virus phylum-level structure (Figure 4D), it can be observed that the virus phylum Uroviricota dominates absolutely (95.29–98.07%). At the genus level, a heatmap of the top 15 abundant genera in the virus communities was constructed (Figure 5D). The heatmap shows that the virus communities in the farmland and wetland soils cluster together, followed by a cluster with grassland soil microbial communities, while the *Tamarix chinensis* and *Fraxinus chinensis* forests cluster together. The analysis of the Shannon diversity index, the Simpson diversity index, the Chao1 index, and the Ace index of soil virus communities under five land use types (Figure 6D) shows that the Shannon and Simpson indices of the five land use types do not show significant differences (*p* > 0.05). The Chao1 and Ace indices are highest in the grassland soils, significantly higher than those in the *Tamarix chinensis* forest, *Fraxinus chinensis* forest, farmland, and wetland (*p* < 0.05). Based on the Bray–Curtis distance, the PCoA analysis shows that soil viral communities are differentiated between different land use types (Figure 7D). The PERMANOVA analysis (Table 2) indicates that virus community differences are significant (*p* < 0.05) between all land use types except the *Tamarix chinensis* forest and *Fraxinus chinensis* forest, the *Tamarix chinensis* forest and farmland, the *Tamarix chinensis* forest and wetland, the *Fraxinus chinensis* forest and farmland, and the farmland and wetland.

The Lefse analysis of soil virus communities in different land use types is shown in Figure 8D. At the phylum, class, order, family, and genus classification levels, significant differences in dominant species are found among the five land use types. At the order level, Thumleimavirales in grassland was a significant difference in land use type. At the family level, Kyanoviridae in *Fraxinus chinensis* forest; Autographiviridae in farmland; and Casjensviridae in grassland were significant differences in land use types. At the genus level, Fipvunavirus, Rimavirus, Bingvirus, Nipunavirus, and Flowerpowervirus in *Tamarix chinensis* forest; Riverridervirus, and Myxoctovirus in *Fraxinus chinensis* forest; Delepquintavirus, Lessievirus, Jasminevirus, Yuavirus, Plateaulakevirus, and Gyeongsanvirus in farmland; Johnsonvirus and Yonginvirus in wetland; and Noxifervirus in grassland were significant differences in land use types.

## 4. Discussion

The soil microbial community plays an extremely important role in ecosystems, particularly in maintaining soil health and promoting plant growth. The community structure and diversity of soil microorganisms directly affect ecological functions such as nutrient cycling, organic matter decomposition, and pollutant degradation. Investigating the relationship between soil microbial communities and ecosystem functions will contribute to the formulation of land management and ecological restoration decisions. For example, a global study by Xu et al. [36], based on 2110 paired observation data from 107 published papers, shows that, depending on the soil conditions, controlling the amount and production conditions of biochar application is a direct way to regulate the impact of biochar on soil microbial functions and enhance the soil carbon storage capacity. The differences in microbial communities among land use types are typically determined by the interaction of various environmental factors, including soil pH, moisture, organic matter content, nutrient levels, soil temperature, salinity, and land management practices. The impact of each land use type on these factors is unique, leading to the formation of distinct microbial community structures. Different land use types alter the physical and chemical properties of the soil, including moisture, nutrient content, and organic matter levels, which in turn affect the structure and function of soil microbial communities [37,38,39]. The study reveals the impact of different land use types on soil microbial community structure and highlights the common ecological patterns within microbial communities. Wetland soils exhibit a higher microbial diversity, particularly in bacterial, eukaryota, archaea, and virus communities. In wetlands, both community abundance and evenness are superior to those in other land use types, which is likely closely related to the unique moisture conditions and organic-rich environment of the wetlands. The redox gradients and complex ecological niches in wetlands make them an ideal habitat for the coexistence of a variety of microorganisms. In contrast, farmland, due to frequent tillage, fertilization, and chemical interventions, shows a lower microbial diversity and a more uniform community structure. Forests (*Tamarix chinensis* and *Fraxinus chinensis* forests), on the other hand, promote the proliferation of specific microorganisms due to organic matter accumulation and carbon sources from plant root exudates, particularly in the archaeal and fungal communities, which exhibit a relatively higher diversity. Overall, environmental factors such as soil moisture, organic matter content, and plant types play a decisive role in shaping the microbial community structure.

### 4.1. Characteristics of the Soil Bacteria Community Structure in Different Land Use Types

At the phylum level of bacteria communities, Proteobacteria, Acidobacteria, and Actinobacteria are the most common phyla. This finding is consistent with previous research on soil bacteria, indicating that these three phyla dominate most soil bacterial communities. For example, a study by Zhang et al. [40] on wetlands along the lower reaches of the Songhua River highlighted that, with changes in land use, Proteobacteria, Acidobacteria, and Actinobacteria became the primary dominant taxa. Similarly, research by Chen et al. [41] in the Loess Plateau also confirmed that these three phyla dominate soil bacterial communities in that region. More importantly, there is the relative abundance of these bacterial phyla changes under different land use types. For instance, the abundance of Proteobacteria is highest in grassland soils, while Acidobacteria dominates wetland soils. These differences may reflect the impact of soil physicochemical properties and nutrient conditions on the microbial community composition. A heatmap analysis shows that the bacteria community structure in *Tamarix chinensis* and *Fraxinus chinensis* forest is relatively similar, while farmland and wetland soils display larger differences, which are closely related to the differences in microbial niches under different land uses. The diversity indices of bacteria communities (the Shannon and Simpson indices) are significantly higher in wetland soils compared with other land uses, while the Chao1 and Ace indices are lower in farmland and wetland soils, indicating a higher microbial richness and evenness in wetland soils. This is consistent with previous studies [42], as wetlands, with better moisture conditions and more complex environments, provide more ecological niches in anaerobic conditions, fostering the coexistence of various microorganisms. In contrast, the bacteria community diversity in farmland soils is lower, which may be closely related to frequent tillage, fertilization, and chemical treatments that lead to a more simplified microbial community. In *Fraxinus chinensis* forest, the dominant bacteria groups are related to organic matter degradation and carbon–nitrogen cycling. The Myxococcota phylum, which is highly abundant, produces various hydrolytic enzymes involved in biopolymer degradation processes and plays a crucial role in the ecosystem [43,44]. Other dominant groups, such as Actinomycetota, Acidobacteria, and Planctomycetes, also play important roles in carbon–nitrogen cycling [45,46,47], with Acidobacteria having cellulose-degrading capabilities [9]. In farmland soils, Sphingomonadales, with its significant potential in pesticide degradation and nitrogen fixation, is the dominant group, suggesting its considerable ecological role in environmental protection and farmland production [48,49].

### 4.2. Characteristics of the Soil Eukaryota Community Structure in Different Land Use Types

Ascomycota dominates the eukaryota communities in all land use types, consistent with previous findings [50,51]. Ascomycota eukaryota are diverse, with many species being plant pathogens, decomposers, or symbiotic eukaryota, especially those involved in decomposing plant litter. These eukaryota play an essential ecological role in the decomposition of plant litter, effectively converting organic material into usable nutrients, thus maintaining nutrient cycling and replenishment in the soil [52]. Therefore, the high abundance of Ascomycota in soil eukaryota communities is related not only to the richness of soil organic matter but also to its functional role in the ecosystem. Notably, in *Fraxinus chinensis* forest soils, Ascomycota shows significant dominance, which could be due to the accumulation of organic matter promoted by root exudates and litter, which may be the primary reason for the increased abundance of Ascomycota. In *Tamarix chinensis* forest soils, the Shannon diversity index reaches the highest value, with the Chao1 and Ace indices significantly higher than those in farmland and wetland soils (*p* < 0.05), indicating a higher species diversity and community evenness in eukaryota communities. This is in line with the findings of Gomez et al. [53], where abundant plant litter in forested areas provides rich organic nutrients for soil eukaryota, promoting their growth and enhancing eukaryota diversity. The presence of mycorrhizal eukaryota, which form symbiotic relationships with plant roots, further increases eukaryota diversity. These eukaryota help plants to acquire water and minerals from the soil, particularly in nutrient-poor or dry environments, and in return, the plants provide organic carbon and nutrients to the eukaryota, thus boosting eukaryota growth and diversity. This mutualistic relationship plays an important role in stabilizing the soil structure and facilitating nutrient cycling in ecosystems [54].

### 4.3. Characteristics of the Soil Archaea Community Structure in Different Land Use Types

The study of soil archaea communities revealed that Nitrososphaerota dominated the archaea community with an abundance ranging from 74.93% to 82.81%, which is consistent with the findings from other studies. Nitrososphaerota plays a significant role in soil nitrogen cycling, particularly in ammonia oxidation, making it a critical participant in the nitrogen cycle [8]. Its higher abundance in farmland soils indicates that farmland soils have stronger pesticide degradation and nitrogen cycling functions. Furthermore, Euryarchaeota, with an abundance of 10.09–16.31%, also plays a significant role, particularly in methane generation during the carbon cycle [55]. At the genus level, significant differences in archaea communities were found across land uses. A heatmap analysis revealed that farmland and grassland soils share high similarities in archaea communities, as do *Tamarix chinensis* and *Fraxinus chinensis* forest soils, possibly due to similarities in plant types and soil environments [56]. These community similarities are further reflected in diversity index differences, where grassland soils exhibit significantly higher Shannon, Simpson, Chao1, and Ace indices compared with farmland soils (*p* < 0.05); the differences between these forests and other land uses were highly significant (*p* < 0.05), which aligns with the genus-level analysis results. The Lefse analysis revealed significant dominant species of soil archaea communities under different land use types. At the phyla level, Euryarchaeota in *Fraxinus chinensis* forest and Nitrososphaerota in farmland were significantly different phyla. At the class level, Methanomicrobia in *Fraxinus chinensis* forest and Halobacteria in wetland showed a strong predominance. Studies have shown that the distribution of these dominant species in different environments is closely related to soil nitrogen and carbon cycles and microbial metabolic functions [57]. In addition, at the genus level, Methanoperedens and Nitrosocosmicus in *Tamarix chinensis* forest occupy a dominant position in the community, reflecting that this area may have high methanogenesis and nitrogen oxidation activities.

### 4.4. Characteristics of the Soil Virus Community Structure in Different Land Use Types

This study also analyzed soil virus communities under different land use types, revealing that land use types significantly affect the virus community structure. At the phylum level, Uroviricota dominated across all land use types, with the relative abundance ranging from 95.29% to 98.07%, consistent with previous studies on soil virus communities [58]. Other phyla, such as Nucleocytoviricota and Preplasmiviricota, had lower relative abundances, which may be due to the specific host requirements or ecological adaptations of these viruses [59]. At the genus level, significant structural differences were observed in soil virus communities under different land uses. A heatmap analysis showed that virus community structures in farmland and wetland soils were more similar, suggesting that these two land uses may share similar ecological conditions, leading to the similarity in the virus community structure. *Tamarix chinensis* and *Fraxinus chinensis* forest soils also showed some similarity in virus community clustering, likely due to the specific microenvironmental conditions in forest ecosystems, such as soil moisture and organic matter content. These results further indicate that different land uses indirectly regulate the soil virus community structure by affecting environmental factors such as pH, temperature, and organic matter content. The diversity indices of virus communities showed that grassland soils had a significantly higher diversity than other land uses, particularly in the Chao1 and Ace indices. This could be attributed to the richer plant community and more complex microecological environment in the grassland. In contrast, *Tamarix chinensis* forest, *Fraxinus chinensis* forest, farmland soils, and wetland had lower diversity indices, likely due to more homogeneous soil conditions and plant species. A PCoA analysis confirmed the significant differentiation of virus communities across land uses, validating the impact of land use types on the virus community structure [60]. Among the different land use types, the viral communities showed obvious differences in phyla, class, order, family, and genus taxonomic levels, particularly at the genus level, where *Tamarix chinensis* forest virus genera, such as Fipvunavirus and Rimavirus, *Fraxinus chinensis* forest genera, such as Riverridervirus and Myxoctovirus, and grassland genera, such as Noxifervirus, exhibited significant differences. These specific virus genera are likely related to particular environmental factors, such as the soil type, vegetation, and microclimate. Future research could explore the relationship between these viruses and their hosts, as well as their potential roles in ecosystem functions.

Microbial biotechnology in agriculture holds great potential. By applying biotic fertilizers, biopesticides, and bioremediation techniques, it can effectively enhance soil health, increase crop yields, and promote sustainable development [61]. Incorporating microbial diversity monitoring into conservation or agricultural policies can effectively assess the impact of land use on ecosystems. Through monitoring microbial communities, conservation policies can identify key species and promote ecological restoration, while agricultural policies can optimize farming practices, reduce chemical interventions, and improve soil health and agricultural sustainability. Although this study provides preliminary insights on the impact of land use on microbial community structure and diversity, there are still some shortcomings and limitations that warrant further exploration and refinement in future research. One major limitation is the lack of in-depth investigation into how specific land use factors influence the microbial metabolism or community interactions. Future studies could integrate techniques such as genomics, transcriptomics, and metabolomics to further analyze the impact of land use changes on microbial function, and longitudinal observations could be employed to reveal the long-term dynamics of microbial communities. Additionally, the absence of time-series data and comprehensive measurements of environmental factors (such as soil temperature, salinity, humidity, etc.) represents another gap in this study. Future research should consider collecting data at different time points and incorporate environmental factors for a more comprehensive analysis to better understand the driving mechanisms of microbial community changes. Finally, it is recommended that future studies include longitudinal analyses and experimental manipulations of land use to explore the long-term effects of different land management practices, validate the patterns observed in this study, and clarify the potential mechanisms underlying land use changes in microbial communities.

## 5. Conclusions

This study reveals the structural characteristics and diversity changes of soil microbial communities under different land use types of the Huanghe alluvial plain. Firstly, land use significantly influenced the composition of soil microbial communities, with distinct structural features in soil bacteria, eukaryota, archaea, and virus communities under different land use types. Bacteria communities were primarily dominated by Proteobacteria, Acidobacteria, and Actinobacteria; eukaryota communities by Ascomycota, Zygomycota, and Basidiomycota; archaea communities by Nitrososphaerota and Euryarchaeota; and virus communities by Caudoviruses. Secondly, different land uses had a significant impact on microbial diversity, with higher species diversity observed in wetland and grassland soils. The Lefse analysis further identified significant indicator species for each land use type, which could serve as potential biomarkers to assess the impact of different land use types on soil ecosystems. The results of this study demonstrate that the establishment of diverse habitats on abandoned farmland can significantly enhance soil microbial diversity, with the creation of wetland ecosystems leading to higher levels of microbial diversity in the soil. This research provides important microbial ecological insights for land use decision-making in the Yellow River alluvial plain, contributing to the sustainable use of soil resources and ecological conservation in the region. Incorporating microbial diversity monitoring into conservation or agricultural policies can effectively assess the impact of land use on ecosystems. By monitoring microbial communities, conservation policies can identify key species and promote ecological restoration, while agricultural policies can optimize farming practices, reduce chemical interventions, and enhance soil health and agricultural sustainability. This approach contributes to advancing the transition to sustainable agriculture and ensures the long-term stability of the ecological environment.

## Figures and Tables

**Table 1:** Distribution of soil microorganisms.

	TCF	FCF	FL	WL	GL
Bacteria	90.64%	90.94%	92.45%	91.50%	91.90%
Eukaryota	0.75%	0.58%	1.01%	0.78%	0.98%
Archaea	0.01%	0.01%	0.01%	0.01%	0.01%
Virus	0.04%	0.02%	0.07%	0.06%	0.09%

**Table 2:** PERMANOVA tests for the adonis of soil bacteria, eukaryota, archaea, and virus.

Microbial Taxa	Land Use Types	F-Value	R^2^	*p*-Value	Adjust *p*-Value
Bacteria	TCF vs. FCF	1.65	0.17	0.103	0.103
	TCF vs. FL	6.64	0.45	0.005	0.015
	TCF vs. WL	4.89	0.38	0.02	0.022
	TCF vs. GL	11.74	0.59	0.008	0.015
	FCF vs. FL	9.58	0.54	0.012	0.015
	FCF vs. WL	5.85	0.42	0.01	0.015
	FCF vs. GL	17.71	0.69	0.007	0.015
	NT vs. WL	6.68	0.45	0.007	0.015
	NT vs. GL	11.53	0.59	0.011	0.015
	WL vs. GL	5.52	0.41	0.012	0.015
Eukaryota	TCF vs. FCF	2.02	0.2	0.009	0.014
	TCF vs. FL	3.1	0.28	0.008	0.014
	TCF vs. WL	3.56	0.31	0.011	0.014
	TCF vs. GL	6.97	0.47	0.012	0.014
	FCF vs. FL	4.77	0.37	0.015	0.015
	FCF vs. WL	5.56	0.41	0.013	0.014
	FCF vs. GL	12.05	0.6	0.007	0.014
	NT vs. WL	2.77	0.26	0.010	0.014
	NT vs. GL	4.43	0.36	0.010	0.014
	WL vs. GL	3.53	0.31	0.004	0.014
Archaea	TCF vs. FCF	3.04	0.28	0.097	0.097
	TCF vs. FL	6.76	0.46	0.008	0.013
	TCF vs. WL	3.84	0.32	0.008	0.013
	TCF vs. GL	24.6	0.75	0.008	0.013
	FCF vs. FL	14.41	0.64	0.009	0.013
	FCF vs. WL	3.00	0.27	0.045	0.050
	FCF vs. GL	23.86	0.75	0.011	0.014
	NT vs. WL	6.07	0.43	0.009	0.013
	NT vs. GL	29.06	0.78	0.008	0.013
	WL vs. GL	4.26	0.35	0.007	0.013
Virus	TCF vs. FCF	1.29	0.14	0.286	0.318
	TCF vs. FL	1.86	0.19	0.132	0.165
	TCF vs. WL	2.03	0.20	0.106	0.151
	TCF vs. GL	11.21	0.58	0.010	0.035
	FCF vs. FL	2.48	0.24	0.060	0.100
	FCF vs. WL	3.03	0.27	0.027	0.054
	FCF vs. GL	14.84	0.65	0.012	0.035
	NT vs. WL	0.84	0.09	0.507	0.507
	NT vs. GL	3.45	0.30	0.008	0.035
	WL vs. GL	3.50	0.30	0.014	0.035

## Data Availability

The data are not publicly available due to privacy.
